# Long-term cost-effectiveness of health behaviour intervention to manage type 2 diabetes in Nepal

**DOI:** 10.1186/s12916-025-03981-8

**Published:** 2025-03-11

**Authors:** Padam Kanta Dahal, Corneel Vandelanotte, Lal Rawal, Rashidul Alam Mahumud, Grish Paudel, Melanie Lloyd, Yeji Baek, Biraj Karmacharya, Tomohiko Sugishita, Zanfina Ademi

**Affiliations:** 1 School of Health, Medical and Applied Sciences, Central Queensland University, Sydney Campus, 400 Kent Street, NSW Rockhampton, 2000 Australia; 2 Appleton Institute, Physical Activity Research Group, Central Queensland University, Queensland, Australia; 3 Translational Health Research Institute (THRI), Western Sydney University, Sydney, NSW Australia; 4 NHMRC Clinical Trials Centre, Faculty of Medicine and Health, The University of Sydney, Camperdown, NSW Australia; 5 Health Economics and Policy Evaluation Research (HEPER) Group, Centre for Medicine Use and Safety, Faculty of Pharmacy and Pharmaceutical Sciences, Monash University, Melbourne, Australia; 6 School of Public Health and Preventive Medicine, Monash University, Melbourne, Australia; 7 Department of Community Medicine, Kathmandu University Hospital, Dhulikhel, Nepal; 8 Section of Global Health, Department of Hygiene and Public Health, Tokyo Women’s Medical University, Tokyo, Japan

**Keywords:** Type 2 diabetes, Long-term cost-effectiveness, Markov modelling, Health behaviour intervention

## Abstract

**Background:**

Long-term cost-effectiveness analyses of health behaviour interventions to effectively manage type 2 diabetes mellitus (T2DM) in low-income countries are crucial for minimising economic burden and optimising resource allocation. Therefore, this study aimed to estimate the long-term cost-effectiveness of implementing a health behaviour intervention to manage T2DM in Nepal.

**Methods:**

A Markov model in combination with a decision tree was developed to compare the costs and outcomes of the health behaviour intervention against usual care among 481 (238-intervention and 243-control) participants from healthcare system and societal perspectives. The model integrates empirical trial data, with published data to inform parameters not collected during the trial. The model estimated costs, quality-adjusted life years (QALYs) and cost-effectiveness over 5 years, 10 years, 20 years, 30 years and a lifetime time horizons with 3% annual discounting. Sub-group, scenarios, both one-way and two-way analyses and probabilistic sensitivity analyses (PSA) were performed to assess the impact of uncertainty in the model under the threshold of 3 times gross domestic product (GDP) per capita (i.e., US $4140) for Nepal.

**Results:**

Base-case analysis with lifetime horizon showed that the health behaviour intervention compared to usual care improved QALYs by 3.88 and increased costs by US $4293 per patient, with an incremental cost-effectiveness ratio (ICER) of US $1106 per QALY gained from a healthcare system perspective. From a societal perspective, QALYs also improved by 3.88 and costs increased by US $4550, with an ICER of US $1173 per QALY gained. Furthermore, the intervention demonstrated ICERs of US $636, US $678, US $637, and US $632 per QALY gained over 5-, 10-, 20-, and 30-year time horizons, respectively, from a healthcare system perspective, and US $719, US $766, US $659, and US $716 per QALY gained from a societal perspective*.* In the PSA, the probability of the health behaviour intervention being cost-effective was over 57%.

**Conclusions:**

The health behaviour intervention for managing T2DM was cost-effective over a lifetime horizon compared to usual care. To maximise its impact, this intervention should be scaled up nationwide, and future research is warranted to assess the long-term cost-effectiveness across diverse settings in low-income countries.

**Trial registration:**

Australia and New Zealand Clinical Trial Registry (ACTRN12621000531819).

**Graphical Abstract:**

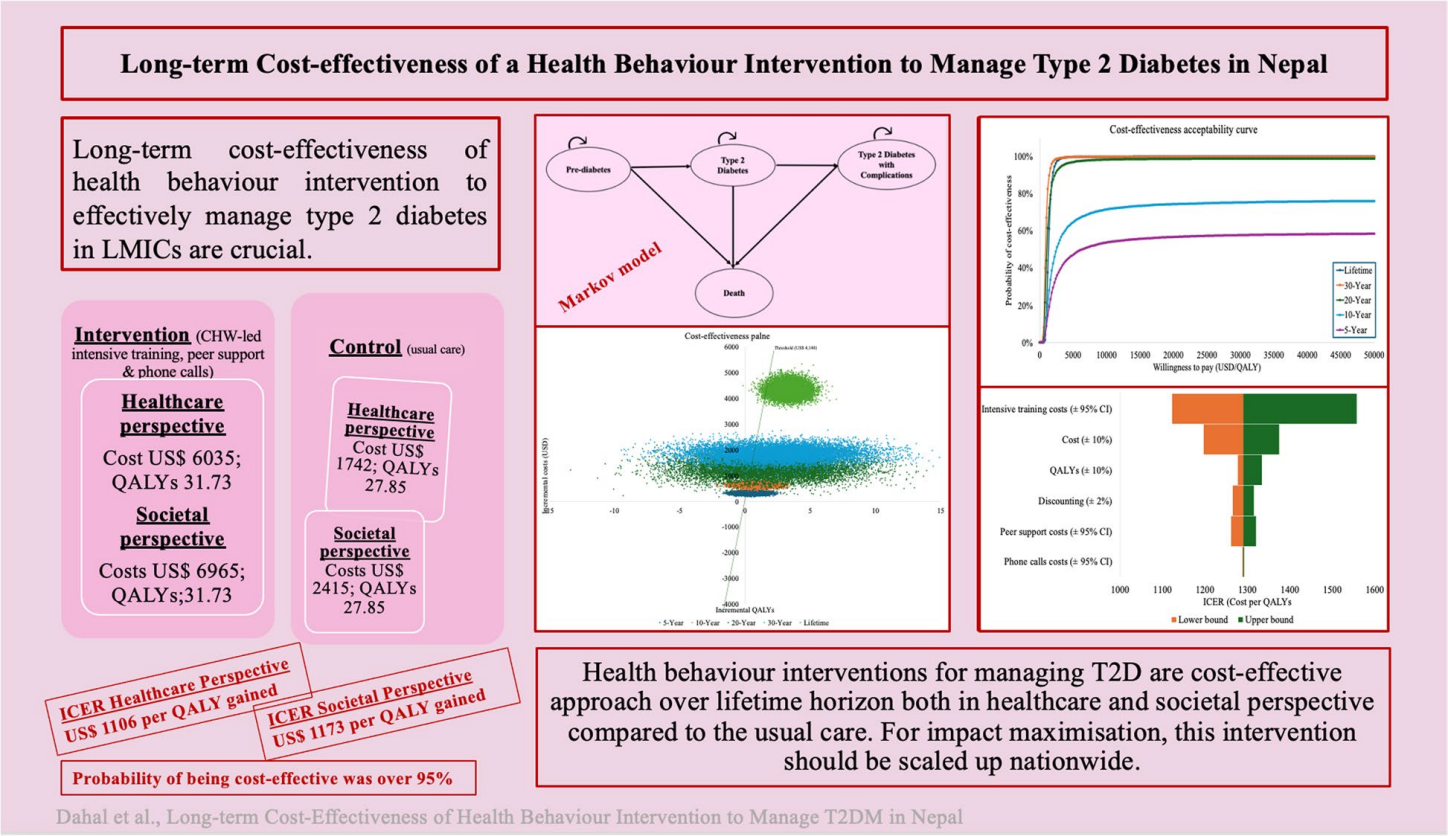

**Supplementary Information:**

The online version contains supplementary material available at 10.1186/s12916-025-03981-8.

## Background

Type 2 diabetes mellitus (T2DM) is a global public health problem, with a substantial financial burden to the healthcare systems which is projected to be more than doubled by 2050 [1]. The International Diabetes Federation (IDF) estimated that total healthcare expenses of US $966 billion were incurred in the year 2021 and are projected to reach US $1.05 trillion by 2045 [2]. These expenses are expected to rise dramatically in low and lower-middle-income countries (LMICs) like Nepal, where prevalence is projected to increase from 8.7% in 2021 to 9.4% by 2045 [2]. Further, many cases remain undiagnosed in Nepal due to the financial barriers and limited healthcare services for diagnosis and treatment [3]. Total expenses in Nepal are anticipated to increase to US $190.5 million, and US $168.1 per person by the year 2045 which is almost double the 2021 estimates [2].

Health behaviour interventions have been shown to be highly cost-effective for managing T2DM and its complications over the longer term [4–8]. The modelling study of the Da Qing Diabetes Prevention Program in China predicted that its health behaviour intervention was cost-effective, with cost-savings of US $818 (¥5338) per patient over 30 years and US $294 (¥1,921) over a lifetime time horizon [9]. A simulation model of a nationwide, community-based health behaviour intervention within the US healthcare system estimated cost-savings of US $5.7 billion within 25 years [10]. Furthermore, systematic reviews of health behaviour interventions in high-income countries indicated that interventions intended to prevent T2DM are cost-effective or cost-saving in the longer term [11–13]. However, this evidence is poorly generalisable to LMICs like Nepal where healthcare resources are limited and the economic burden of diabetes is increasing [14–17]. Therefore, the long-term impact of health behaviour interventions in resource poor settings remains unknown for decision-makers. To address this knowledge gap, this study aimed to evaluate the long-term (i.e., 5-year, 10-year, 20-year, 30-year and lifetime time horizons) cost-effectiveness of community-based health behaviour intervention compared to usual care to manage type 2 diabetes in Nepal by using a decision tree in combination with Markov modelling from both healthcare system and societal perspectives.

## Methods

This study is a model-based health economic evaluation using clinical trial data from a health behaviour intervention compared to usual care to manage T2DM in Nepal and integrates published data to inform parameters not directly collected during the trial. The study was conducted from both a Nepalese healthcare system and societal perspectives. The components of this intervention include intensive training on diabetes self-management led by community health workers (CHW), peer supporters and phone calls. The intervention study consists of a sample of 481 participants (i.e., intervention (n = 238) and control group (n = 243)) aged 30–70 years who are clinically diagnosed with T2DM and that have the ability to respond to a health behaviour intervention. Participants were recruited from 30 randomly selected clusters from two selected districts (Kavrepalanchok and Nuwakot) in Nepal. These clusters were randomised into intervention and control groups (15 in each group) where participants were automatically allocated to the cluster based on their residency [18]. The clinical trial was registered in the Australia and New Zealand Clinical Trial Registry (ACTRN1262100053181) and reporting of this economic evaluation follows the 2022 Consolidated Health Economic Evaluation Reporting Standards (CHEERS) (Additional file 1: Table S1) [19].

### Intervention

The details of the health behaviour intervention study have been described elsewhere [18]. In brief, in addition to the existing care, a combination of intensive training on diabetes self-management led by CHW, peer supporters and regular telephone calls (fortnightly calls for the initial 3 months and monthly calls thereafter) were provided to the intervention participants for six months. The intensive training comprised 12 modules of diabetes self-management practices including physical activity, dietary adherence, drinking alcohol and smoking cessation, healthcare utilisation, medication adherence, footcare, regular blood sugar monitoring, oral health, quality of life, stress management, complication reduction strategies and social and emotional support, accompanied by a pictorial book on diabetes management. The control group received usual care alongside the pictorial book for diabetes self-management.

### Markov model structure

**Fig. 1:**
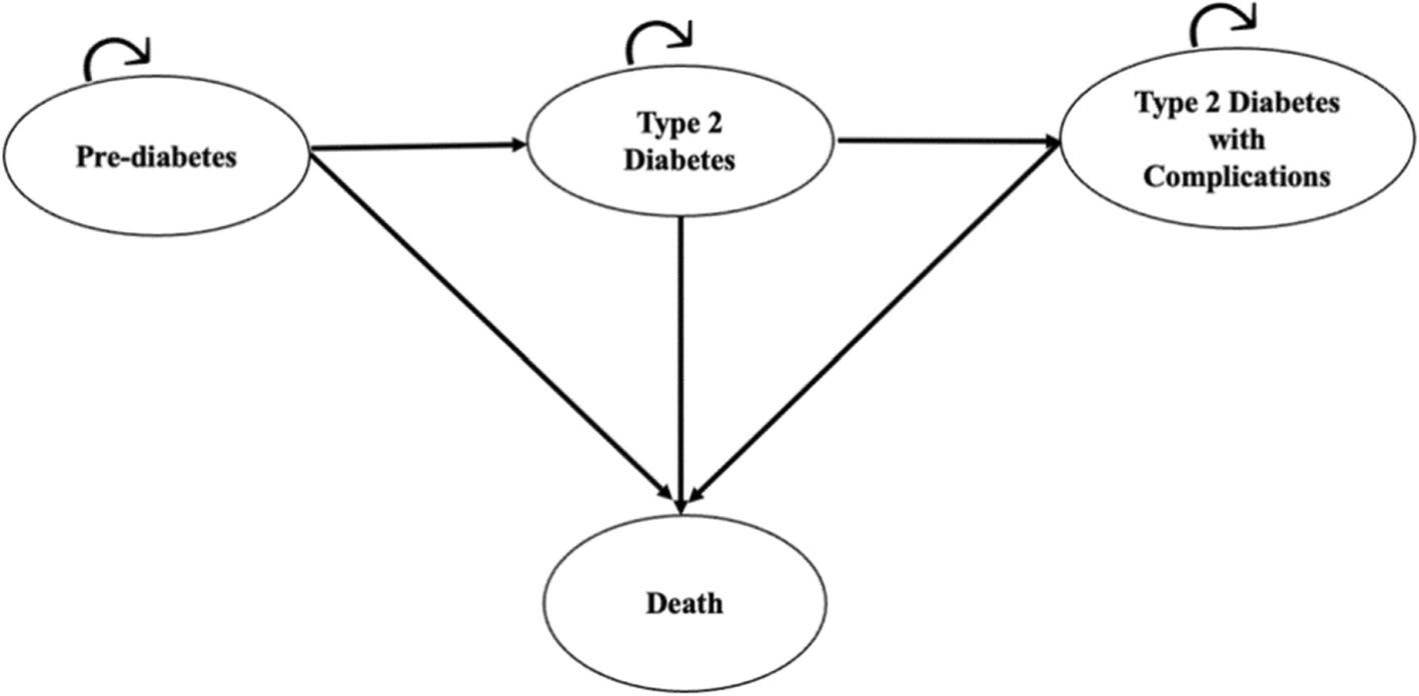
Schematic diagram of Markov model with all arrows indicative of possible transition probabilities

We constructed a decision tree in combination with a Markov model. The model compares the health and downstream economic consequences of intervention vs. usual care over a lifetime time horizon. The model consists of four health states (1) pre-diabetes; (2) T2DM (3) T2DM with complications and (4) death. The pre-diabetes health state includes individuals who had been diagnosed with a HbA1c level between 5.7%−6.4%. Further, individuals in this health state may have elevated blood glucose levels and are at risk of developing type 2 diabetes. After an annual cycle, individuals can remain in the same health state or move to the T2DM health state (i.e., state 2), T2DM with complications (i.e., state 3) or death (i.e., state 4). Individuals in health state 2 have progressed from pre-diabetes and were characterised by blood sugar levels (i.e., HbA1c level 6.5% to 9%) and may require specific interventions to manage their condition. Individuals in health state 2 can stay in the same state or move to state 3 or 4. In state 3, individuals may have diabetic complications such as cardiovascular diseases, diabetic retinopathy, nephropathy, neuropathy and other related complications with HbA1c greater or equal to 9%. Individuals can stay in this same state or die (i.e., state 4). State 4 is an absorbing health state (Fig. 1).

### Transition probabilities

The probability of moving from one health state to another at the end of each cycle is illustrated through transition probabilities. This model considered eight transition probabilities within four different health states. These probabilities were extracted from previous studies and converted to annual transition probabilities by using the formula (i.e., p = 1- exp(-rt)), where p is transition probability, r is the disease occurrence rate and t is the time frame (Table 1) [20, 21]. The transition probability of participants in state one in the intervention arm suffering from possible type 2 diabetes conditions and complications was regarded as a baseline probability. The estimated prevalence of pre-diabetes in Nepal was determined to be 19.4% based on a recent systematic review study [22]. The remaining patients were distributed across other health states in accordance with their respective proportions, which were used to establish the initial conditions for the analysis.

**Table 1:** Input parameters for the cost-effectiveness analysis

Input parameters	Intervention	Control	Distribution	Sources
Age in Mean (SD)	54.26 (9.12)	54.62 (9.71)	Log-normal	[26]
Transition probability				
Pre-diabetes to T2DM (1 to 2)	0.04	0.10	Normal	[25]
Pre-diabetes to death (1 to 4)	0.014	0.014	Normal	[27]
T2DM to complication (2 to 3)^a^	0.13	0.34	Normal	[26, 28]
T2DM to death (2 to 4)	0.004	0.02	Normal	[26]
Complication** to death (3 to 4)^a^	0.01	0.01	Normal	[26]
Relative risk				
Pre-diabetes	0.46	na	Normal	[25]
T2DM	0.88	na	Normal	[29]
Mortality (all cause)	Life table			
Cost per patient (US $) *				
Total costs	126.04	68.46	Gamma	[26]
Total intervention	34.64	na	Gamma	[26]
Intensive training	28.66	na	Gamma	[26]
Peer support	5.04	na	Gamma	[26]
Phone calls	0.94	na	Gamma	[26]
Patient income loss	25.66	18.22	Gamma	[26]
Medical consultation	8.32	7.70	Gamma	[26]
Screening	28.62	26.86	Gamma	[26]
Medication	18.70	7.36	Gamma	[26]
Hospitalisation	5.60	2.90	Gamma	[26]
Utility (mean)*	0.86	0.84	Beta	[26]
Pre-diabetes^a^	0.87	0.87	Beta	[26]
T2DM^a^	0.90	0.86	Beta	[26]
Complications^a^	0.85	0.79	Beta	[26]

^*^Adopted from the six months trial data, costs were doubled to make an annual change

** cardiovascular disease events mortality;

^a^based on the same trial data but calculated separately;

*T2D* Type 2 diabetes, *SD* Standard deviation, *QALYs* Quality-adjusted life years, *RCT* Randomised Control Trial evaluating the current examined health behaviour intervention in Nepal, *na* not applicable

In the intervention group, the annual transition probability of moving from health state one to two, state two to three or four, and state three to four was adopted from the Da Qing Diabetes Prevention Program in China [9] systematic review studies [22–25] and our recent study [26]. These studies were chosen as the source for key input parameters due to their rigorous methodology, relevance to our study population and comprehensive studies addressing the disease progression and intervention effects. Further, the systematic review and meta-analyses went through a quality appraisal of the study design, sample size, population characteristics and outcome consistency to ensure its reliability and applicability to the model. The reported transition rates (i.e., incidence rates, or relative risks) for transitions between health states were converted to annual transition probability. The probabilities of T2DM with complications health state were derived from the baseline value by multiplying the relative risk ratio (i.e., 1.13) of co-morbid conditions (i.e., hypertension, high blood cholesterol, asthma, and heart problems) at the baseline of the current study. Further, the probability of moving from T2DM to T2DM with complications was 0.13 and from T2DM to death was 0.004 which were obtained from the current study. Furthermore, RRs were used to adjust the transition probabilities for the intervention group relative to the control group, thereby quantifying the intervention effect on disease progression when direct trial data were not available. All case mortality rates for this pre-diabetic state were obtained from the Nepal life table published by the World Health Organisation (WHO) [27]. The annual probability of moving from pre-diabetes to T2DM was 0.04 which was obtained from systematic review studies of health behaviour intervention [25].

In the control group, participants with pre-diabetes have a 10% probability of developing T2DM, which is considered as an annual transition probability of moving from pre-diabetes to type 2 diabetes [25]. Similarly, the average annual transition probability from T2DM (State 2) to T2DM with complications (state 3) in the control group was 33.95% [28] and three to four was 0.01% (i.e., derived from our current study) (Table 1 and Additional file 1: Table S2).

### Costs

The base costs were estimated from both healthcare system and societal perspectives. Each health state in the model was assigned total healthcare resource costs. The details of cost estimation methods have been described elsewhere [26, 30]. In brief, the cost from a healthcare system perspective, direct medical costs such as healthcare utilisation (i.e., medical consultation, screening, medication, and inpatient costs), direct non-medical costs (i.e., transportation, food and vegetable consumption) and intervention costs (i.e., intensive training, peer support and phone call costs) were explored based on the six months follow up data. From a societal perspective, the cost of patients’ income loss due to the hospital stay, outpatient department (OPD) visit, and travel time were estimated. The estimated average per patient costs from the recent six-month health behavioural intervention (i.e., US $63.02 in the intervention arm and US $34.23 in the control arm) were doubled to make annual costs (Table 1). Costs were calculated in Nepali rupees and converted to US dollars based on the average exchange rate in 2022 (i.e., US $1 = NRs 125.20) [26, 31].

### Utility and health effects

Health-related quality of life (HRQoL) was explored by using the health utility weight (HUW). HUW was valued from 0–1, where 0 denotes death and 1 denotes perfect health. QALY, was used to quantify the health outcome. QALY was calculated by using the utility score multiplied by time that the participant lives in each health state. In this study, health utilities were derived from EQ-5D-3L tool. It has five dimensions that include mobility, self-care, usual activities, pain/discomfort, and anxiety/depression with three possible responses (no problem, moderate problem and severe problem) [32]. Each participant was assigned a health utility based on the condition of their disease and complications. A Nepal-specific algorithm of the EQ-5D-3L does not exist, therefore, we applied the Indian estimates to calculate the utilities where the reverse crosswalk mapping function was applied [33, 34]. The average utility value (i.e., 0.86 per patient in the intervention group and 0.84 in the control group) of was applied as a base annual utility value for this study [26]. However, distinct utility scores for each health state were derived from the empirical trial data, with assumptions made based on corresponding HbA1c levels (see above section ‘Markov Model Structure’).

### Cost-effectiveness

The main outcome of this economic evaluation was the incremental cost-effectiveness ratio (ICER) in terms of cost per QALY gained over a 5-year, 10-year, 20-year, 30-year and lifetime time horizons [35]. In addition, ICERs were estimated to address the uncertainties and offer decision-makers a comprehensive understanding of outcomes under multiple conditions and sub-groups of individual intervention component such as intensive training led by CHW, peer support and phone calls. The cost-effectiveness threshold of Nepal (US$4140) [36] was calculated based on the WHO-CHOICE project recommendations, i.e. three times the national GDP per capita of US$1380 in 2023 [35]. The use of GDP-based thresholds are controversial in the low-and-lower-middle-income countries like Nepal [37]. However, a country-specific threshold has not been established and is still under debate [38]. As such, we are compelled to rely on a GDP-based threshold for this cost-effectiveness analysis. A three percent discounting per annum was applied to costs and QALYs as recommended for LMIC [35].

### Statistical, scenario and sensitivity analyses

Categorical variables were reported as frequency and percentages; continuous variables were as mean and standard deviations. Costs and QALYs were reported as mean and standard deviations and their incremental values, ICERs, were reported with 95% confidence intervals (CIs).

Multiple scenario analyses were performed to determine the impact of health behaviour interventions under multiple scenarios. Firstly, the reduction in the base care prevalence of pre-diabetes, type 2 diabetes and complications in the model by 10%, followed by an additional 30% and 50% reduction were applied to see the impact of health behaviour intervention over different time periods. These simultaneous reductions in prevalence across all health states may not typically occur in reality. However, these assumptions were made to explore the potential effects on cost-effectiveness. This approach helps to assess how concurrent variations in the prevalence rates of each condition might impact the model’s results. Furthermore, discounting rates were varied by 0%, 4% and 5% to assess the influence of study results with different time preferences.

In one-way analyses, we independently varied the costs and QALYs based on their respective upper and lower 95% confidence intervals. Conversely, in two-way analyses, both costs and QALYs were simultaneously varied across their lower and upper 95% CIs. This is because costs and QALYs are the main primary outcome of interest in our cost-effectiveness analysis. Further, these analyses were performed to provide insights into the stability of conclusions across multiple scenarios.

The probabilistic sensitivity analyses were performed using 10,000 Monte Carlo simulations and illustrated the outcomes in the cost-effectiveness plane of each time horizon. Finally, the impact of uncertainty on the model was plotted on the cost-effectiveness acceptability curve as a probability of cost-effectiveness of the health behaviour intervention in relation to the possible values of willing-to-pay (WTP) thresholds. The model development and analyses including sub-group of individual components of health behaviour intervention (such as intensive training led by CHW, peer supports and phone calls), scenarios, and sensitivity analyses were performed in Microsoft Excel Mac version (Version 16.77 (23,091,003)). The economic model was validated through face validation techniques (i.e., expert review of structure and assumptions), and both internal (i.e., trace testing and consistency checks) and external validation techniques (i.e., comparison with published data) to confirm the appropriateness of the model structure, assumptions and clinical relevance (Additional file 1: Figures S1 and S2) [39].

## Results

### Base case results

The results of the base case analyses are summarised in Table 2. From a societal perspective, across the lifetime horizon, the intervention group had total costs amounting to US $6965 per patient, while the control group incurred costs of US $2415 per patient. The incremental cost, from a societal perspective was US $4550 (95% CI = 3747 to 5352) when comparing study groups.

**Table 2:** Cost-effectiveness results of base case analysis

Time horizons and perspective	Intervention		Control		Incremental		ICER^#^ (95%CI)	PBC^##^ (%)
	Costs in mean* (SD)	QALYs in mean ** (SD)	Costs in mean* (SD)	QALYs in mean** (SD)	Costs (95%CI)	QALYs (95% CI)		
5-Year horizon								
Healthcare system	537 (0.20)	4.64 (0.01)	258 (0.28)	4.20 (0.002)	280 (230 to 329)	0.44 (0.36 to 0.52)	636 (524 to 748)	57.29
Societal	670 (0.20)	4.64 (0.01)	354 (0.28)	4.20 (0.001)	317 (260 to 372)	0.44 (0.36 to 0.53)	719 (592 to 846)	57.47
10-Year horizon								
Healthcare system	1069 (0.82)	9.12 (0.02)	506 (1.19)	8.29 (0.02)	563 (464 to 662)	0.83 (0.68 to 0.99)	678 (558 to 798)	76.93
Societal	1334 (0.82)	9.12 (0.02)	698 (1.19)	8.29 (0.02)	636 (524 to 748)	0.83 (0.68 to 0.99)	766 (631 to 902)	76.31
20-Year horizon								
Healthcare system	2067 (4.44)	17.03 (0.07)	939 (4)	15.26 (0.08)	1128 (929 to 1326)	1.77 (1.46 to 2.12)	637 (525 to 749)	96.26
Societal	2442 (4)	17.03 (0.07)	1275 (4)	15.26 (0.08)	1167 (1049 to 1498)	1.77 (1.46 to 2.12)	659 (592 to 846)	96.14
30-Year horizon								
Healthcare system	2912 (10)	22.97 (0.14)	1263 (8)	20.36 (0.14)	1649 (1358 to 1940)	2.61 (2.15 to 3.12)	632 (520 to 743)	97.97
Societal	3709 (10)	22.97 (0.14)	1840 (8)	20.36 (0.14)	1869 (1539 to 2198)	2.61 (2.15 to 3.12)	716 (589 to 842)	97.95
Lifetime time horizon								
Healthcare system	6035 (39)	31.73 (0.33)	1742 (27)	27.85 (0.29)	4293 (3535 to 5050)	3.88 (3.20 to 4.64)	1106 (911 to 1302)	99.98
Societal	6965 (39)	31.73 (0.33)	2415 (27)	27.85 (0.29)	4550 (3747 to 5352)	3.88 (3.20 to 4.64)	1173 (986 to 1379)	99.98

^*^Cumulative costs per person; ** Cumulative QALYs per person; All costs are in United States Dollar (USD); ^#^Incremental cost-effectiveness ratio (ICER) is presented in costs per QALY gained; CIs: Confident interval; ^***##***^Probability of being cost-effectiveness (PBC) after 10,000 Monte Carlo simulations at given threshold (i.e., US $4,140)

Applying a 30, 20, 10, 5 years’ time horizons, the incremental costs were, US $1869 (95% CI = 1539 to 2198), US $1167 (95% CI = 1049 to 1498), US $636 (95% CI = 524 to 748) and US $317 (95% CI = 260 to 372) respectively. These costs were almost equivalent to the control group costs in their respective time horizons from societal perspective.

From a healthcare system perspective, the intervention group incurred a total cost of US $6035 per patient, while the control group incurred $1742 per patient over the lifetime time horizon. The incremental cost was US $4293 (95% CI = 3535 to 5050) per patient over the lifetime time horizon, followed by US $1649 (95% CI = 1358 to 1940) over 30 years, US $1128 (95% CI = 929 to 1326) over 20 years, US $563 (95% CI = 464 to 662) over 10 years, and US $280 (95% CI = 230 to 329) over five years.

From both healthcare system and societal perspectives, the incremental QALYs gained per patient were 3.88 over the lifetime time horizon, 2.61 over 30 years, 1.77 over 20 years, 0.83 over 10 years, and 0.44 over five years. As such, the health behaviour intervention was found to be cost-effective from both healthcare system and societal perspectives.

**Fig. 2:**
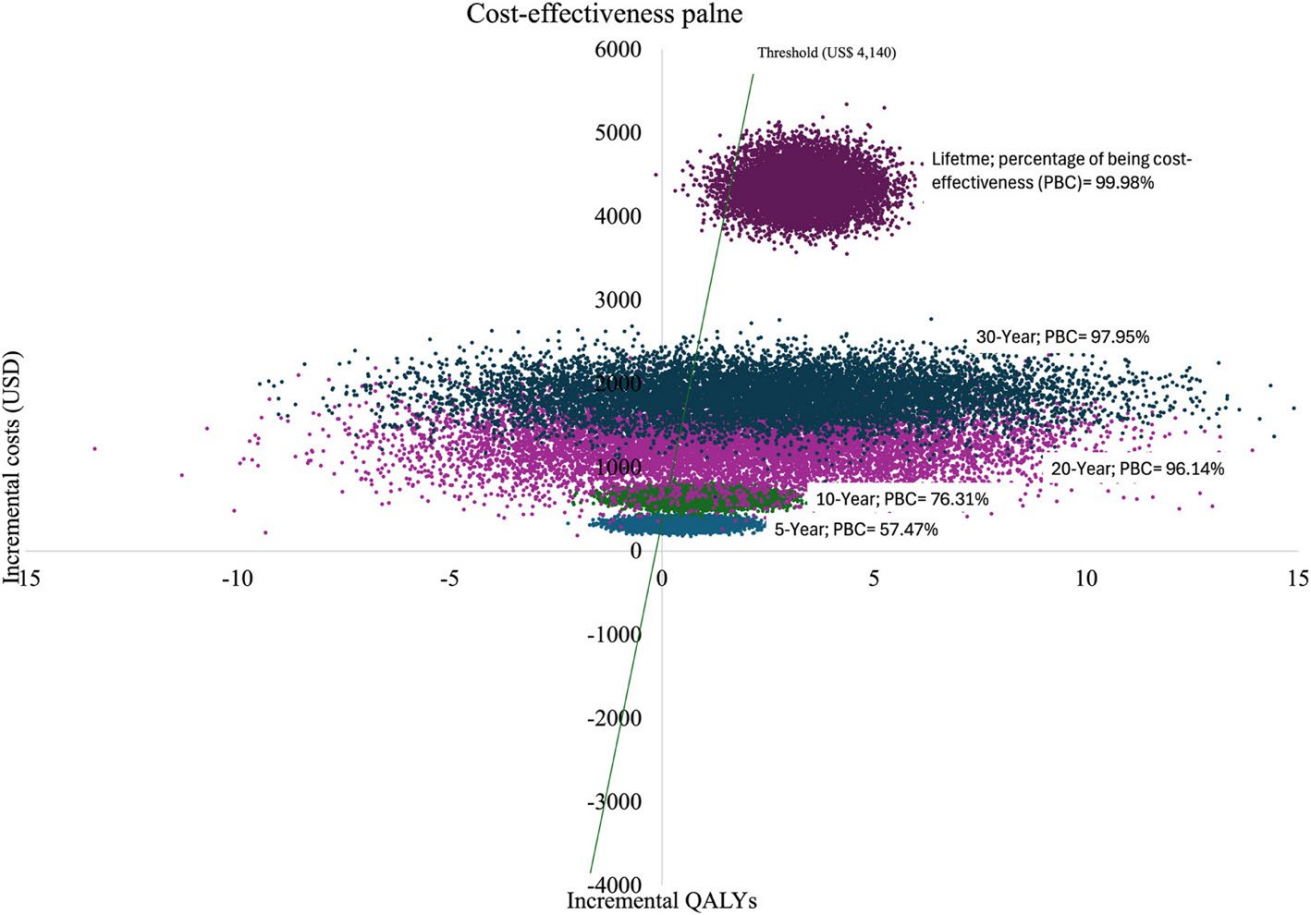
Cost-effectiveness plane of 10,000 Monte Carlo simulations from a societal perspective across multiple time horizons

**Fig. 3:**
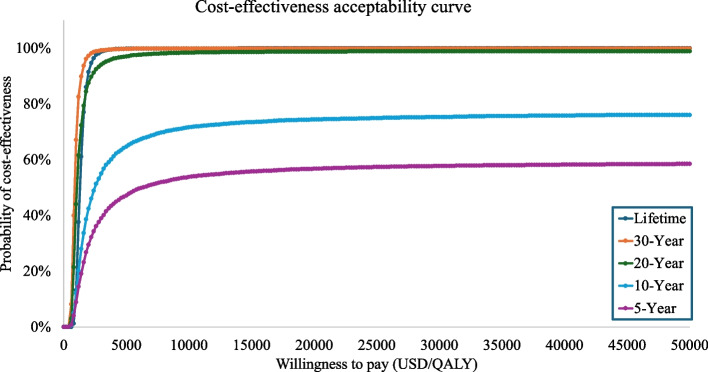
Cost-effectiveness acceptability curve (CEAC) representing multiple horizons from a societal perspective

The probabilistic sensitivity analysis demonstrated that the probability of the health behaviour intervention being cost-effective was over 57%, with a willingness to pay threshold of US $4140 across different time horizons (See Fig. 2 for societal perspective and Additional file: Figure S3 and S4 for the healthcare system perspective). Furthermore, the health behaviour intervention was nearly 100% cost-effective compared to the control across various maximum willingness-to-pay thresholds in the lifetime time horizon (see Fig. 3 for the societal perspective and Additional file 1: Figure S5 for the healthcare system perspective).

### Sub-group, scenarios and sensitivity analysis results

The results of the sub-group, scenarios and sensitivity analyses from a societal perspective are presented in Table 3 and Additional file 1: Table S3. Sub-group analyses showed that implementation of health behaviour change components separately (i.e., CHW-led intensive training sessions, peer support, and regular phone calls) demonstrated cost-effectiveness across all time horizons. However, ICER of intensive training in a five-year time horizon was US $6458 per QALY (95% CI = 5318 to 7597) which is above the threshold value assumed for this study, indicating a lack of cost-effectiveness.

**Table 3:** Sensitivity analysis over the 5, 10, 20, 30 years and lifetime time horizon of health behaviour intervention from a societal perspective

**Categories**	**5-Year horizon**	**10-Year horizon**	**20-Year horizon**	**30-Year horizon**	**Lifetime time horizon**
	ICER (95%CI)*	ICER (95%CI)*	ICER (95%CI)*	ICER (95%CI)*	ICER (95%CI)*
Sub-group					
Peer support	3505 (2887 to 4123)	1291 (1063 to 1518)	528 (435 to 622)	422 (347 to 496)	427 (352 to 503)
Intensive training	6458 (5318 to 7597)	2364 (1947 to 2781)	962 (792 to 1132)	778 (641 to 915)	1130 (931 to 1329)
Phone call	2992 (2464 to 3519)	1104 (909 to 1299)	452 (372 to 532)	358 (295 to 421)	287 (237 to 338)
Scenario-I					
Base case prevalence −10%	9608 (7913 to 11,302)	2759 (2273 to 3246)	1110 (914 to 1306)	888 (731 to 1044)	1313 (1082 to 1545)
Base case prevalence −30%	9601 (7907 to 11,295)	3046 (2508 to 3583)	1212 (998 to 1425)	949 (782 to 1116)	1375 (1132 to 1617)
Base case prevalence −50%	14,398 (11,858 to 16,937)	3852 (3172 to 4531)	1380 (1136 to 1623)	1038 (855 to 1221)	1475 (1215 to 1735)
Scenario-II					
Undiscounted	7421 (6111 to 8730)	2597 (2138 to 3055)	1073 (884 to 1262)	866 (713 to 1018)	1291 (1063 to 1518)
Discounting 4%	2962 (2439 to 3484)	2857 (2353 to 3361)	2806 (2311 to 3301)	2876 (2379 to 3384)	2879 (2371 to 3386)
Discounting 5%	2908 (2395 to 3421)	2918 (2403 to 3432)	2936 (2418 to 3454)	2904 (2392 to 3416)	2858 (2354 to 3362)
One-way					
Costs (Lower CI)	5793 (4771 to 6815)	2119 (1745 to 2493)	861 (709 to 1012)	696 (573 to 819)	1037 (854 to 1220)
Costs (Upper CI)	8617 (7097 to 10,137)	3152 (2596 to 3708)	1280 (1054 to 1506)	1036 (853 to 1218)	1543 (1271 to 1815)
QALY (Lower CI)	9606 (7912 to 11,301)	3222 (2653 to 3790)	1341 (1105 to 1578)	1077 (887 to 1266)	1606 (1322 to 1889)
QALY (Upper CI)	−930 (−656 to −1093)	2230 (1837 to 2624)	891 (734 to 1048)	724 (596 to 851)	1078 (888 to 1268)
Two-way					
Costs and QALY (Lower CIs)	7724 (6361 to 9086)	2590 (2133 to 3047)	1078 (888 to 1268)	866 (713 to 1019)	1291 (1063 to 1519)
Costs and QALY (Upper CIs)	−1112 (−916 to −1308)	2667 (2180 to 3114)	1065 (877 to 1253)	865 (713 to 1019)	1289 (1062 to 1517)

*QALYs *Quality Adjusted Life Years, *CIs* Confident Intervals*,* *ICER Incremental Cost-effectiveness Ratio (Presented in cost per QALY gained), *T2D* Type 2 Diabetes

In scenario I, the health behaviour intervention was not cost-effective over a five-year time horizon. However, it became cost-effective as the time horizon extended to 10 years, 20 years, 30 years and a lifetime. Similarly, in scenario II, the health behaviour intervention was cost-effective in all conditions (0%, 4%, and 5% discounting), except in the five-year time horizon without discounting, where the ICER was US $7421 per QALY, indicating that it was not cost-effective.

**Fig. 4:**
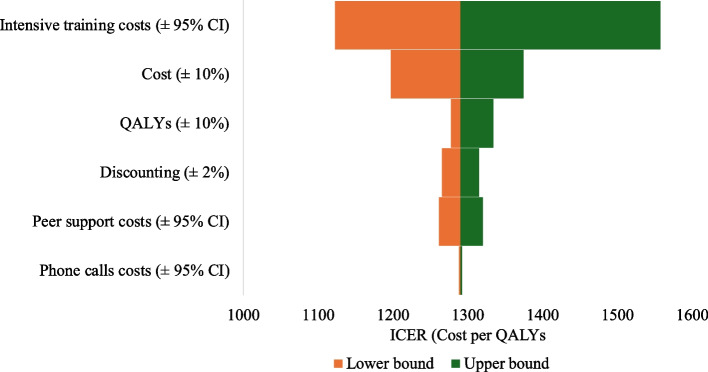
Tornado analysis of ICERs in base case analysis over the lifetime horizon from a societal perspective as an example

In addition, the tornado diagram of the base case analysis indicates that changes in intensive training costs by ± 95% CI have the largest impacts on ICERs (i.e., almost US $268/QALYs in the upper bound and US $157/QALYs in the lower bound) followed by changes in total costs and QALYs (± 10%) over the lifetime time horizons (see Fig. 4 for the societal perspective and Additional file 1: Figure S6 for the healthcare system perspective).

Both one-way and two-way sensitivity analyses illustrated health behaviour intervention was cost-effective, with an associated ICER of less than US $3222 per QALY gained over 10-Year time horizons.

## Discussion

To our knowledge, this is the first study to evaluate the long-term economic impact of health behaviour intervention to manage T2DM in Nepal. Our study found that the implementation of combined health behaviour intervention for managing T2DM was cost-effective across the lifetime time horizon. Further, this intervention was cost-effective and has the potential to be highly cost-effective compared to usual care, even when multiple different subgroups, scenarios, time horizons and perspectives are considered.

Our study indicated that while the intervention improved T2DM outcomes, healthcare costs are likely to be more than double than those of usual care. Over the lifetime time horizon, the costs in the intervention group (US $6965) were more than triple than those in the control group (US $2415), which is due to additional healthcare resource utilisation including medication use, specialist visits, early diagnosis of possible complications, and hospitalisation. Similar findings were observed in several other comparable studies [40–43]. For example, a modelling study of health behaviour intervention through regular physical activity, healthy diet and weight reduction in Swedish community setting in 2017 showed that intervention costs and QALYs gained were higher compared to control [42]. Similarly, a lifetime simulation model of a patient empowerment program through health behaviour change (i.e., an education program on self-management, behaviour modification and coping mechanism) in Hong Kong by Lian et al., in 2018 reported that cost and QALY gained by intervention group were higher by US $115 and 0.24 per patient, respectively [43]. Hence, this rise in costs and effects might be attributed to the intervention’s aims to increase access to and utilisation of healthcare services to manage T2DM. This might be an indication of positive effects on lifestyle change by optimum use of available healthcare resources. However, a study in a health resource-limited setting in China in 2020 by Ma et al., predicted that total costs among the control group were higher by 6.5% and QALYs were lesser compared to intervention over a lifetime horizon [44]. This might be due to the patients in the control group experiencing more adverse health events during the study period, leading to increased healthcare utilisation and costs. As such, despite variation in costs, the offset by increases in effects (i.e., QALYs) underscores the potential value of health behaviour interventions in improving health outcomes.

The health behaviour intervention proved cost-effective in managing T2DM in a low resource setting over long-term time horizons. For instance, our current study identified ICERs (i.e., ranges of US $636 per QALY gained – US $1173 per QALY gained) fourfold lower than the given threshold, indicating the highly cost-effectiveness nature of health behaviour intervention compared to usual care across various long-term time horizons. This key finding is consistent across sub-group, scenario and sensitivity analyses over ten years to lifetime time horizons. In contrast, a systematic review found that behavioural interventions for most high income settings remain cost-effective within the shorter and longer timeframe, possibly due to differences in healthcare resource and cost-effectiveness thresholds [45]. These settings often have dedicated budgets, more efficient healthcare delivery systems, and different willingness-to-pay thresholds, which allow interventions to demonstrate cost-effectiveness within a shorter timeframe. In contrast, in low- and middle-income settings, longer timeframes may be needed to realise the full economic benefits due to limited resources and the slower impact of interventions. Further key findings of our study are comparable to the Da Qing Diabetes Prevention Program in China, where six years of health behaviour change interventions were proven to be cost-effective and cost-savings over 30 years and lifetime time horizons [9]. However, ICER over the lifetime horizon was five times lower than our estimates, which might be due to the significant impact of interventions such as efficient resource utilisation. Hence, health behaviour interventions provide more health benefits compared to usual care, indicating highly cost-effective strategies [46]. Additionally, a systematic review focused on Asian countries found that health behaviour interventions to manage T2DM were more cost-effective over longer time frames compared to shorter ones [47]. Hence, implementing culturally tailored health behaviour interventions in the low-resource community contexts proves to be cost-effective over an extended period in managing T2DM.

Our study is the first to our knowledge to apply local community-based data to estimate the long-term cost-effectiveness of health behaviour intervention across multiple assumptions and scenarios in Nepal. This new evidence provides valuable insights for Nepali decision-makers and offers a preliminary assessment of the applicability of overseas data to the Nepali population while acknowledging that further validation with locally available data is needed to reduce uncertainties. Further, the costs and utility measured were adopted from a recent intervention which could be helpful in achieving a more accurate estimate of both costs and effects. In addition, some effects and transition probabilities were obtained from meta-analyses and studies of similar settings that are representative of the Nepali population.

There are some important limitations of our modelling study. Firstly, our model assumed four different health states and multiple associated transitions which may not fully capture the dynamic nature of disease progressions (e.g., population from pre-diabetes to complication state and movement from the T2DM state or the T2DM with complications state, to their preceding states). Secondly, our input parameters particularly costs and effects were obtained from the six-month trial data and converted to annual transitions. This duration might not provide sufficient time to assess the fullest impact of the health behaviour intervention on costs and QALYs, potentially influencing our modelling outcomes. Further, we failed to account the seasonal influence on costs and QALYs. Thirdly, the findings from the societal perspective might lack generalisability due to missing data on absenteeism and presenteeism, which could affect accurate estimations of long-term cost-effectiveness from a societal viewpoint. Finally, the model is limited to T2DM with complications, where we generalise the overall complication rates obtained from the previous review. This approach may not cover all the micro and macrovascular complications of T2DM and related transitions, potentially influencing the findings of the modelling process.

Our study holds significant implications for healthcare policy, practice and future research. From a policy standpoint, our study’s findings underscore the importance of optimising healthcare allocations to maximise health outcomes while minimising costs. Policymakers in low-income countries such as Nepal, should prioritise implementing health behaviour change interventions at the national level, aiming for sustained effects and enhance the quality of care for all individuals. Healthcare practitioners should be encouraged to incorporate these interventions into clinical practices, fostering self-care behaviours, peer support, regular phone communications, and empowering patients through training and motivational initiatives. Furthermore, providing regular training and incentives to the community healthcare workers could reinforce the consistent utilisation of health behaviour interventions in practice. Ultimately, our study suggests that more research should be conducted to evaluate the sustainability of health behaviour change interventions in low-income country settings, such as Nepal.

## Conclusions

In conclusion, the implementation of health behaviour intervention for managing T2DM proved highly cost-effective in the longer term, benefiting both the healthcare system and society at large. This intervention exhibits notable improvements in health outcomes, as evidenced by increased QALYs over extended time horizons. However, future research needs to assess the long-term cost-effectiveness and sustainability of health behaviour change interventions across diverse settings, particularly in low-income countries like Nepal. Such studies are essential for informing evidence-based policy decisions and ensuring the continued efficacy of interventions aimed at combating T2DM and improving health outcomes.

## Supplementary Information

Additional file 1: Tables S1-S2, Figures S1-S5, Table S3 and Figures S6. Table S1: Consolidated Health Economic Evaluation Reporting Standards 2022statement. Table S2: Input parameters from the clinical trials for the model development. Figure S1: Markov model trace for control arm across lifetime horizons from a societal perspective. Figure S2: Markov model trace for intervention arm across lifetime horizons from a societal perspective. Figure S3: Percentage of being cost-effectiveacross different time horizons of base case analyses from both healthcare and societal perspectives. Figure S4: Cost-effectiveness plane of 10,000 Monte Carlo simulations from a healthcare perspective across multiple time horizons. Figure S5: Cost-effectiveness acceptability curverepresenting multiple horizons from a healthcare perspective. Table S3: Additional sensitivity analyses with support of literature-based data across lifetime horizons. Figure S6: Tornado analysis of ICERs in base case analysis over the lifetime horizon from a healthcare perspective. 

## Data Availability

No datasets were generated or analysed during the current study.

## Abbreviations

T2DM: Type 2 diabetes mellitus
LMICs: Low and lower middle-income countries
QALYs: Quality adjusted life years
CHEERS: Consolidated health economic evaluation reporting standards
WHO-CHOICE: World health organisation choosing interventions that are cost-effective
IMF: International monetary fund
GDP: Gross domestic product
ICER: Incremental cost-effectiveness ratio
CHW: Community Health Worker
OPD: Out-patient department
WTP: Willingness to pay
