# Supplementary material for: Long-term cost-effectiveness of health behaviour intervention to manage type 2 diabetes in Nepal
Source: BMC Med. 2025 Mar 11;23:153. doi: 10.1186/s12916-025-03981-8 (PMC11900644; doi:10.1186/s12916-025-03981-8)
Supplement: Supplementary file 1 — Additional file 1: Tables S1-S2, Figures S1-S5, Table S3 and Figures S6. Table S1: Consolidated Health Economic Evaluation Reporting Standards 2022statement. Table S2: Input parameters from the clinical trials for the model development. Figure S1: Markov model trace for control arm across lifetime horizons from a societal perspective. Figure S2: Markov model trace for intervention arm across lifetime horizons from a societal perspective. Figure S3: Percentage of being cost-effectiveacross different time horizons of base case analyses from both healthcare and societal perspectives. Figure S4: Cost-effectiveness plane of 10,000 Monte Carlo simulations from a healthcare perspective across multiple time horizons. Figure S5: Cost-effectiveness acceptability curverepresenting multiple horizons from a healthcare perspective. Table S3: Additional sensitivity analyses with support of literature-based data across lifetime horizons. Figure S6: Tornado analysis of ICERs in base case analysis over the lifetime horizon from a healthcare perspective. [file 12916_2025_3981_MOESM1_ESM.pdf]

Additional file 1:

Table S1: Consolidated Health Economic Evaluation Reporting Standards 2022 (CHEERS) statement

Topic	No.	Item	Location where item is reported
Title			
	1	Identify the study as an economic evaluation and specify the interventions being compared.	Page 1
Abstract			
	2	Provide a structured summary that highlights context, key methods, results, and alternative analyses.	Page 1-3
Introduction			
Background and objectives	3	Give the context for the study, the study question, and its practical relevance for decision making in policy or practice.	Page 3-4
Methods			
Health economic analysis plan	4	Indicate whether a health economic analysis plan was developed and where available.	Page 4-7
Study population	5	Describe characteristics of the study population (such as age range, demographics, socioeconomic, or clinical characteristics).	Page 4-5
Setting and location	6	Provide relevant contextual information that may influence findings.	Page 4
Comparators	7	Describe the interventions or strategies being compared and why chosen.	Page 4-7
Perspective	8	State the perspective(s) adopted by the study and why chosen.	Page 4
Time horizon	9	State the time horizon for the study and why appropriate.	Page 5 and 10
Discount rate	10	Report the discount rate(s) and reason chosen.	Page 11
Selection of outcomes	11	Describe what outcomes were used as the measure(s) of benefit(s) and harm(s).	Page 8-11
Measurement of outcomes	12	Describe how outcomes used to capture benefit(s) and harm(s) were measured.	Page 8-12
Valuation of outcomes	13	Describe the population and methods used to measure and value outcomes.	Page 11
Measurement and valuation of resources and costs	14	Describe how costs were valued.	Page 9
Currency, price date, and conversion	15	Report the dates of the estimated resource quantities and unit costs, plus the currency and year of conversion.	Page 9
Rationale and description of model	16	If modelling is used, describe in detail and why used. Report if the model is publicly available and where it can be accessed.	Page 5-7
Analytics and assumptions	17	Describe any methods for analysing or statistically transforming data, any extrapolation methods, and approaches for validating any model used.	Page 11-12
Characterising heterogeneity	18	Describe any methods used for estimating how the results of the study vary for subgroups.	Page 11-12

Characterising distributional effects	19	Describe how impacts are distributed across different individuals or adjustments made to reflect priority populations.	Page 11
Characterising uncertainty	20	Describe methods to characterise any sources of uncertainty in the analysis.	Page 11-12
Approach to engagement with patients and others affected by the study	21	Describe any approaches to engage patients or service recipients, the general public, communities, or stakeholders (such as clinicians or payers) in the design of the study.	Page 5-6
Results			
Study parameters	22	Report all analytic inputs (such as values, ranges, references) including uncertainty or distributional assumptions.	Page 12
Summary of main results	23	Report the mean values for the main categories of costs and outcomes of interest and summarise them in the most appropriate overall measure.	Page 12-15
Effect of uncertainty	24	Describe how uncertainty about analytic judgments, inputs, or projections affect findings. Report the effect of choice of discount rate and time horizon, if applicable.	Page 12-16
Effect of engagement with patients and others affected by the study	25	Report on any difference patient/service recipient, general public, community, or stakeholder involvement made to the approach or findings of the study	Page 12-16
Discussion			
Study findings, limitations, generalisability, and current knowledge	26	Report key findings, limitations, ethical or equity considerations not captured, and how these could affect patients, policy, or practice.	Page 17-21
Other relevant information			
Source of funding	27	Describe how the study was funded and any role of the funder in the identification, design, conduct, and reporting of the analysis	Page 22
Conflicts of interest	28	Report authors conflicts of interest according to journal or International Committee of Medical Journal Editors requirements.	Page 22

Source: Husereau D, Drummond M, Augustovski F, et al. Consolidated Health Economic Evaluation Reporting Standards 2022 (CHEERS 2022) Explanation and Elaboration: A Report of the ISPOR CHEERS II Good Practices Task Force. Value Health 2022;25. doi:10.1016/j.jval.2021.10.008.

Table S2: Input parameters from the clinical trials for the model development

Parameter	Intervention	Control
Age in Mean (SD) [a]	54.26 (9.12)	54.62 (9.71)
Statewise transition probabilities		
Pre-diabetes to T2DM (1 to 2)[b]	0.04	0.10
Pre-diabetes to death (1 to 4)[c]	0.014	0.014
T2DM to complication (2 to 3)[a, d]	0.13	0.34
T2DM to death (2 to 4)[a]	0.004	0.021
Complications to death (3 to 4)[a]	0.01	0.01
Relative risk		
Pre-diabetes[b]	0.46	-
T2DM[e]	0.88	-
Complications	1.13	-
Utility values (mean) [a]	0.86	0.84
Pre-diabetes (HbA1c level <6.5%)[a]	0.87	0.87
T2DM (HbA1c level 6.5% to 8.99%)[a]	0.86	0.86
Complication (HbA1c level ≥9%)[a]	0.85	0.79
Cost per patient (US $) [a]		
Total costs [a]	126.04	68.46
Total intervention [a]	34.64	-
Intensive training [a]	28.66	-
Peer support [a]	5.04	-
Phone calls [a]	0.94	-
Patient income loss [a]	25.66	18.22
Medical consultation [a]	8.32	7.70
Screening [a]	28.62	26.86
Medication [a]	18.70	7.36
Hospitalisation [a]	5.60	2.90

[a] calculated separately from the main trial and informed by Dahal PK, Ademi Z, et al. Health economic evaluation alongside randomised clinical trial of a health behaviour intervention to manage type 2 diabetes in Nepal. Global Health Research and Policy. 2024;9(1):52.), [b]Data was informed by Glechner A, Keuchel L, et al. Effects of lifestyle changes on adults with prediabetes: A systematic review and meta-analysis. Primary Care Diabetes. 2018;12(5):393-408. [c]World Health Organisation. Life table by country Nepal. https://apps.who.int/gho/data/view.main.LT62120?lang=en (2020). [d]Gyawali B, Ferrario A, et al. Challenges in diabetes mellitus type 2 management in Nepal: a literature review. Glob Health Action. 2016;9:31704. [e]Thankappan KR, Sathish T, et al. A peer-support lifestyle intervention for preventing type 2 diabetes in India: A cluster-randomized controlled trial of the Kerala Diabetes Prevention Program. PLoS Med. 2018;15(6):e1002575.

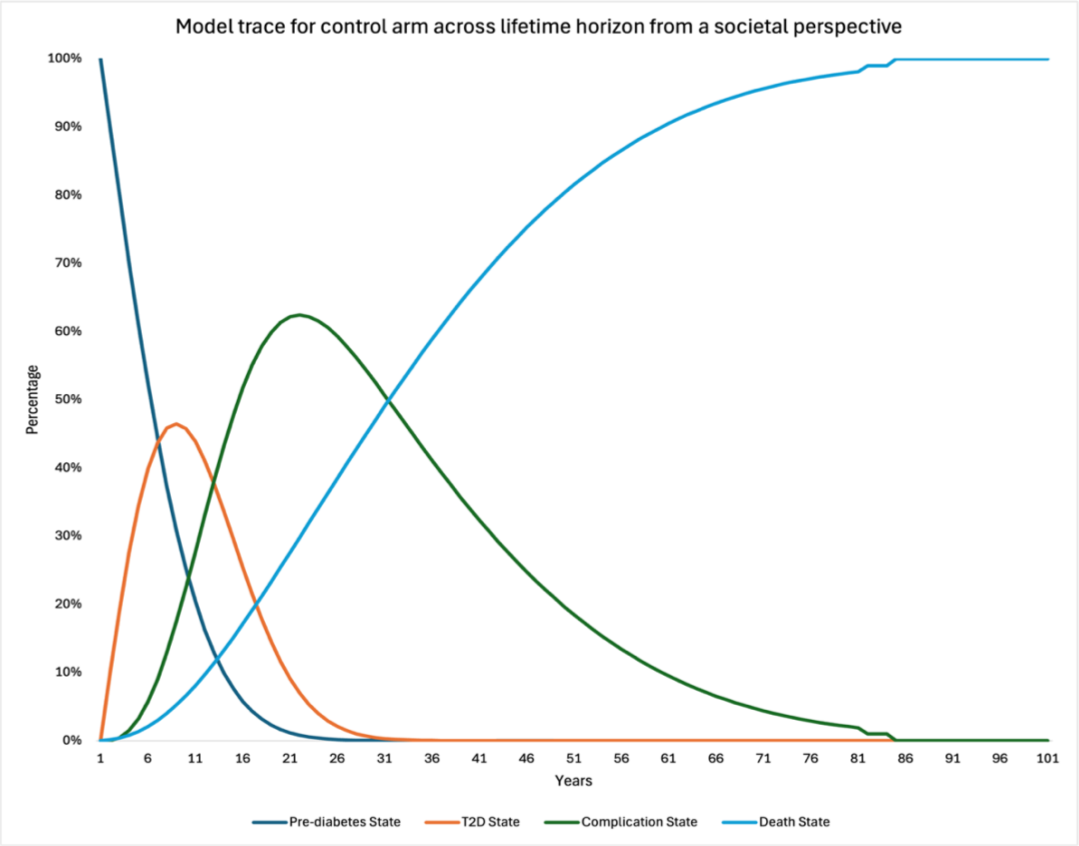

Figure S1: Markov model trace for control arm across lifetime horizons from a societal perspective

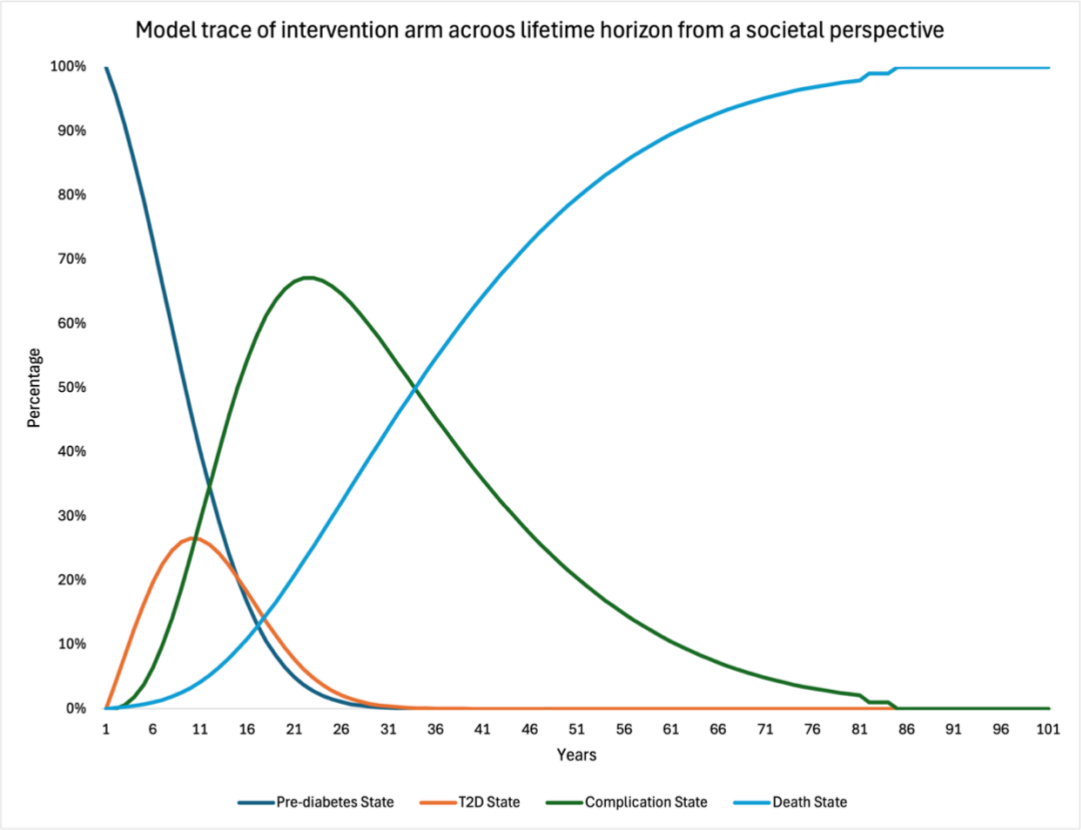

Figure S2: Markov model trace for intervention arm across lifetime horizons from a societal perspective

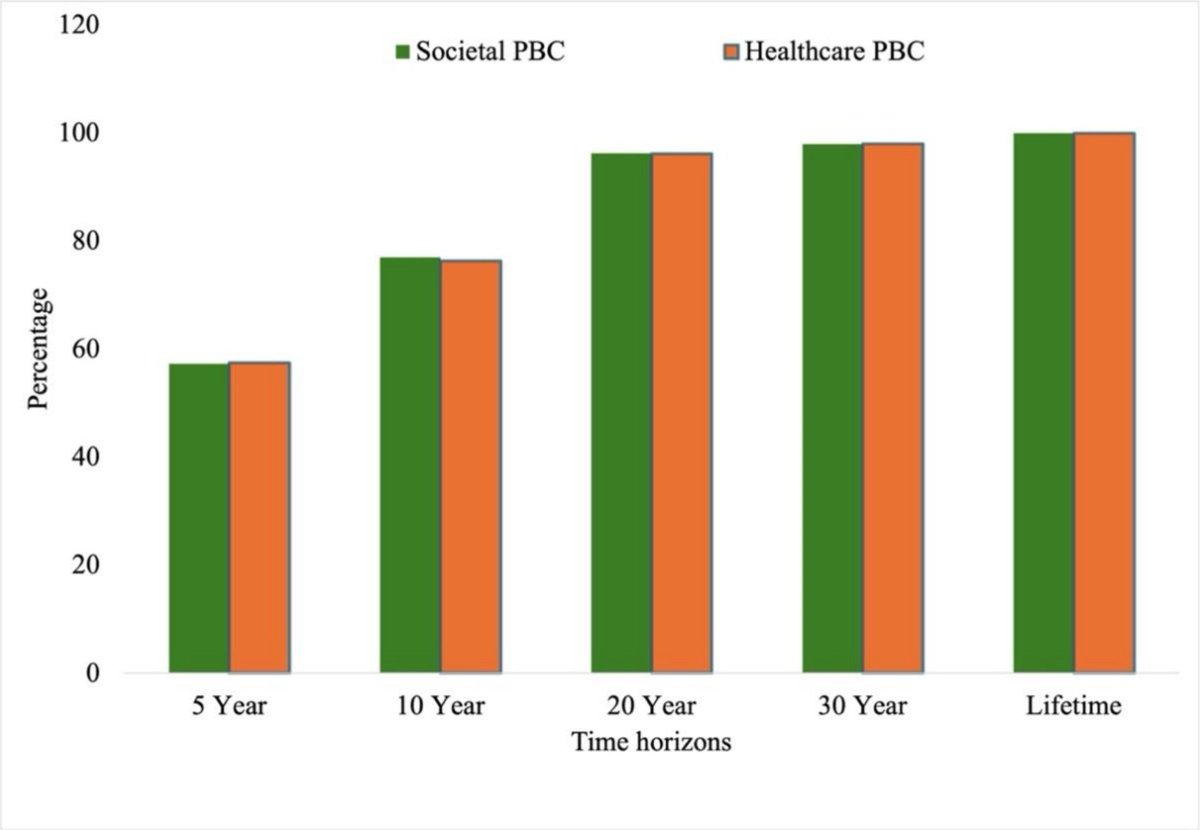

Figure S3: Percentage of being cost-effective (PBC) across different time horizons of base case analyses from both healthcare and societal perspectives

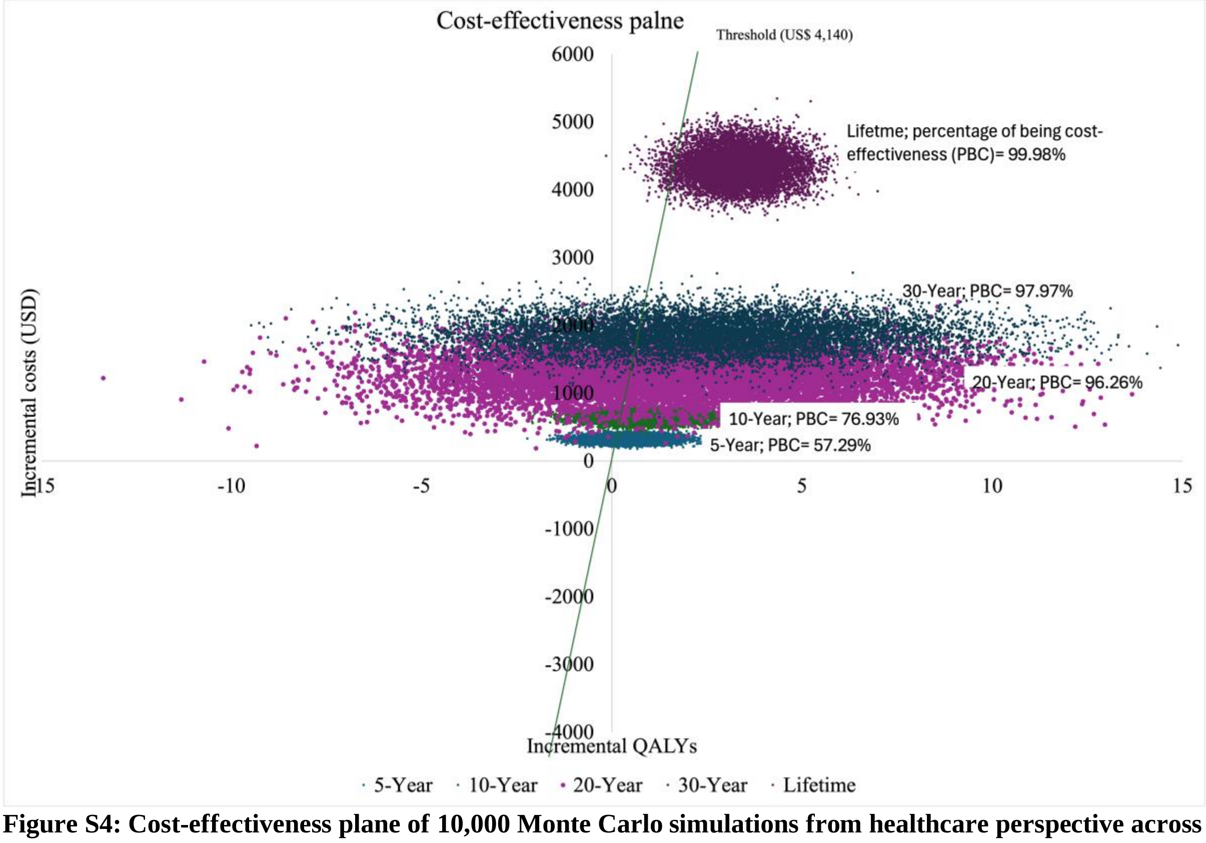

Figure S4: Cost-effectiveness plane of 10,000 Monte Carlo simulations from healthcare perspective across multiple time horizons

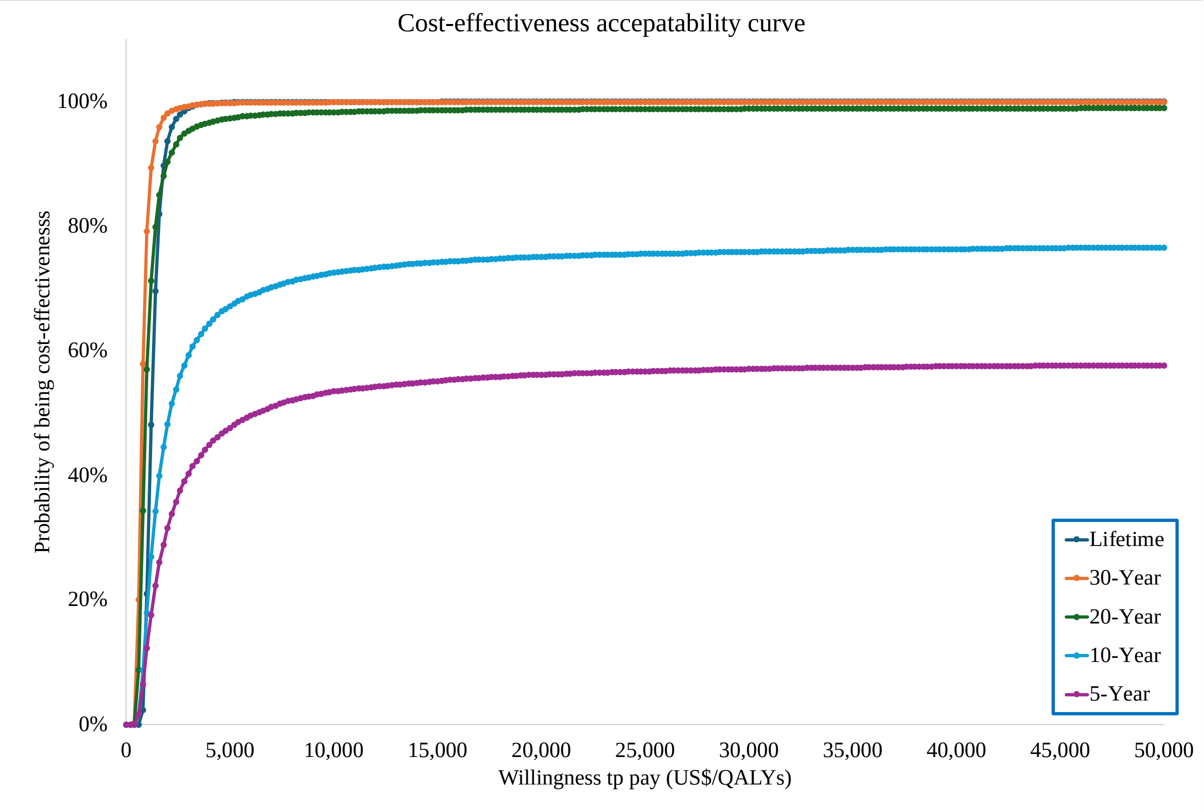

Figure S5: Cost-effectiveness acceptability curve (CEAC) representing multiple horizons from a healthcare perspective

Table S3: Additional sensitivity analyses with support of literature-based data across lifetime horizons

Base case setting	Scenario setting	Incremental cost (US $)	Incremental QALYs	ICER (US $ per QALY gained)	ICER % change from base case
Base case outcome					
	Healthcare	4293.00	3.88	1106.00	-
	Societal	4550.00	3.88	1173.00	-
Health sate utility from the CoLID-Nepal, RCT	Health sate utilities sourced from Hu et. al., 2020*(6)				
	Healthcare	4074.90	1.86	2185.54	97.61
	Societal	4335.30	1.86	2325.21	98.23
3% discountiong	0 % discountiong				
	Healthcare	4415.69	3.92	1212.63	9.64
	Societal	5060.72	3.92	1291.00	10.06
	5% discounting				
	Healthcare	1150.80	0.42	2760.00	149.55
	Societal	1191.67	0.42	2858.00	143.65
Combined health behaviour intervention such as intensive training, phone calls and peer support	Intensive training only				
	Healthcare	3673.11	3.43	1071.10	-3.16
	Societal	3775.11	3.34	1130.00	-3.67
	Phone call only				
	Healthcare	693.21	4.02	172.34	-84.42
	Societal	1071.91	3.73	287.00	-75.53
	Peer support only				
	Healthcare	1383.09	4.27	324.24	-70.68

		Societal	1662.58	3.89	427.00	-63.60
Direct and indirect costs	Exclusion on indirect cost					
		Healthcare	4464.92	4.36	1023.25	-53.18
		Societal	3959.81	4.39	901.37	-23.16
Starting age 30 years	Starting age 60 years					
		Healthcare	3718.34	1.35	2756.64	149.24
		Societal	3755.54	1.35	2784.22	137.36

* This study shares similar population characteristics, and its model states align with our study. While their model includes five health states, we mapped their IGT, T2D, and CVD states to our pre-diabetes, T2D, and complications states, using their utility values

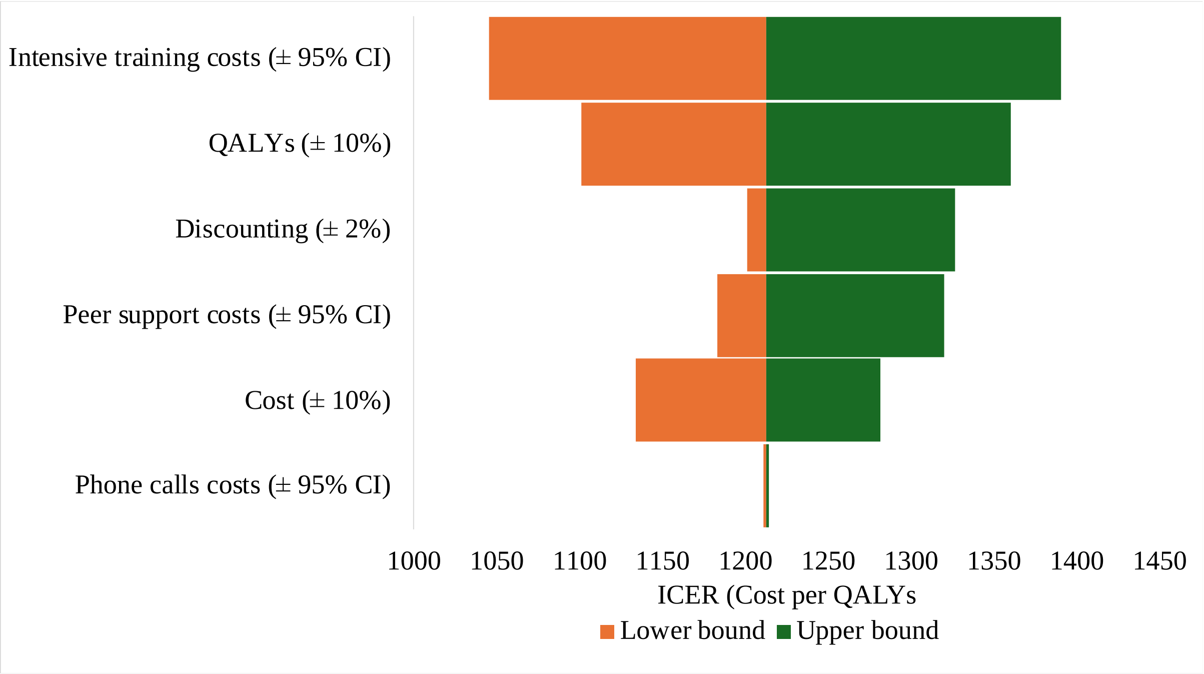

Figure S6: Tornado analysis of ICERs in base case analysis over the lifetime horizon from a healthcare perspective
